# A Challenging Case of Persisting Hypokalemia Secondary to Gitelman Syndrome

**DOI:** 10.7759/cureus.18636

**Published:** 2021-10-10

**Authors:** Mohamad Bakir, Hossam Aldin G Ibrahim

**Affiliations:** 1 Medicine and Surgery, College of Medicine, Alfaisal University, Riyadh, SAU; 2 Internal Medicine/Nephrology, Prince Mohammed Bin Abdulaziz Hospital, Riyadh, SAU

**Keywords:** case report, familial hypokalemia-hypomagnesemia, renal salt wasting, hypomagnesemia, hypokalemia, metabolic alkalosis, gitelman syndrome

## Abstract

There are several causes of hypokalemia, including transcellular shift, renal loss, gastrointestinal loss, and decreased oral intake. Sometimes it is challenging to know the source of the problem; however, with detailed history, physical examination, and appropriate laboratory investigations, the physician should be able to narrow down the differentials diagnosis to reach the right one. One of the rare causes of hypokalemia is Gitelman syndrome, which is a salt-losing tubulopathy that manifests as renal potassium wasting, metabolic alkalosis, hypokalemia, hypomagnesemia, hypocalciuria, and hyperreninemic hyperaldosteronism. This disorder is inherited in an autosomal recessive pattern with an incidence of 25 instances per million population. We report a challenging case of persistent hypokalemia in a 30-year-old woman who presented with a history of palpitation, bilateral upper and lower limbs numbness, nausea, diarrhea, and generalized fatigue for three days. After history and physical examination, the patient was diagnosed with an episode of enteritis, and laboratory workups revealed low potassium and magnesium levels, and it was thought that these electrolyte abnormalities were secondary to gastrointestinal loss. Therefore, the patient was mainly treated supportively along with potassium and magnesium replacement. However, after one week of replacement, the patient still had low potassium and magnesium levels in spite of being diarrhea-free, so renal loss was suspected. Urine electrolytes revealed high renal potassium loss with low-normal blood pressure, arterial blood gases revealed metabolic alkalosis with a pH of 7.49 and bicarbonate level of 29 mEq/L. Repeated urine chemistry was done to check for chloride level and turned out to be high, and 24-hour urinary excretion of calcium was very low. Therefore, the patient was diagnosed with Gitelman syndrome and was managed with potassium and magnesium replacements intravenously, and was encouraged to consume a diet rich in these electrolytes. After complete resolutions of the symptoms and correction of potassium and magnesium levels, the patient was discharged home in stable condition.

## Introduction

Gitelman syndrome (GS) is a salt-losing tubulopathy that manifests as renal potassium wasting, metabolic alkalosis, hypokalemia, hypomagnesemia, hypocalciuria, and hyperreninemic hyperaldosteronism [1]. Mutations in the genes encoding the sodium chloride cotransporter (NCCT) and magnesium transporters in the thiazide-sensitive portions of the nephron's distal convoluted tubule (DCT) cause GS [1]. GS is a rare illness with an estimated prevalence of 25 instances per million people. On the other hand, the prevalence in the heterozygous Caucasian population reaches 1% [2]. Tetany affects the majority of GS patients notably during fever or when additional magnesium is lost owing to vomiting or diarrhea, also paraesthesia, particularly in the face is common. In addition, some patients report acute exhaustion that interferes with everyday activities, while others never complain of fatigue. Furthermore, the degree of hypokalemia does not totally explain the severity of fatigue in GS patients and polyuria is usually absent or mild [2]. GS is a tubular disorder that is inherited in an autosomal recessive pattern where there is a mutation in multiple genes that code for chloride, magnesium, and sodium carriers in the distal convoluted tubule, these carriers are responsible for 7% to 10% of the electrolyte absorption. In addition, in GS, the magnesium channels in the duodenal cells are also down-regulated [1]. Despite greater access to genetic testing, the diagnosis of GS is still largely based on clinical symptoms, particularly in underdeveloped countries. In this paper, we present a challenging case of persistent hypokalemia in a 30-year-old woman secondary to GS.

## Case presentation

**Figure 1:**
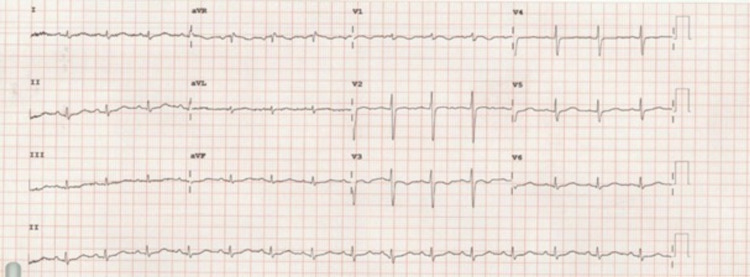
The patient's electrocardiogram (ECG) at the time of admission showing sinus rhythm with prolonged QT interval and U-wave

A 30-year-old woman known case of seasonal bronchial asthma on Ventolin as needed presented with a history of palpitation, bilateral upper and lower limbs numbness, nausea, diarrhea, and generalized fatigue for three days. The palpitation started suddenly while she was sitting, and it continued throughout the day and was not associated with dizziness, syncope, chest pain, or sweating. For the numbness of the limbs, it started first in the upper limbs then it progressed to involve the lower limbs as well and it was associated with generalized fatigue. She had watery diarrhea four times per day, small in amount with no blood, associated with nausea. The patient did not complain of abnormal movement, abdominal pain, or vomiting. There was no history of diuretic use or unusual eating habits. The family history was unremarkable with no kidney or endocrine diseases. She had similar complaints of limbs numbness, and generalized fatigue for the past eight years and she followed up with different hospitals and several investigations were done on her; however, she was never diagnosed or managed appropriately before. On physical examination, the patient was laying on bed conscious, alert and oriented, looks pale, dehydrated, not in respiratory distress. She had intact cranial nerves; the power was five out of five bilaterally in both upper and lower limbs with intact sensation, gait, and coordination. Her vital signs are shown in Table 1. Chest x-ray showed no abnormality, and the electrocardiogram (ECG) showed sinus rhythm with prolonged QT interval, prominent U-wave (Figure 1). Serum electrolytes including, potassium, sodium, magnesium, and chloride were done and showed low serum levels of potassium and magnesium (Table 1). The patient was diagnosed with enteritis and the primary team thought the low potassium and magnesium levels were secondary to the gastrointestinal loss. Therefore, she was given potassium chloride and magnesium sulfate orally but she did not improve so she was placed on potassium chloride 40 mmol twice a day for few days but the serum level of potassium was still on the lower side. For that reason, the nephrology team was consulted for the case and they ordered urine electrolytes and blood gas analysis. The patient was found to have high spot urine potassium and chloride levels. So, 24-hour urine potassium and calcium levels were done and showed high potassium levels along with decreased calcium levels (Table 1). In addition, blood gas analysis showed high pH along with a high bicarbonate level, and the patient was diagnosed with metabolic alkalosis (Table 1). Through these biochemical investigations, we diagnosed the patient with GS after making sure the patient had no reach to thiazide diuretic through a negative thiazide assay. The patient was placed on potassium chloride (80-120 mmol/day) and magnesium sulfate (2-4 g/day) intravenously for few days and was advised to consume foods rich in potassium and magnesium like bananas, dates, dark chocolate, avocados, nuts, and seeds. The patient’s complaints resolved and her electrolyte improved post-treatment (Table 1). The patient did not have any further complaints and she was discharged home in stable condition on magnesium oxide 800 mg and potassium chloride 1,200 mg orally three times per day in addition to spironolactone 50 mg once daily.

**Table 1:** Vital signs and lab values throughout the admission

Vital sign	Patient’s result	Reference range
Temperature	36.8°C	36.6°C to 37°C
Heart rate	121	60-100 beats per minute
Respiratory rate	22	16-20 breaths per minute
Blood pressure	113/72	120/80 mmHg
Oxygen saturation	98 % on room air	
Body mass index	22 kg/m^2^	18.5 kg/m^2^ to 24.9 kg/m^2^
Laboratory test	Patient’s result	Reference range
White blood cells (WBC)	12.1 x 10^9^/L	4-10 x 10^9^/L
Red blood cell (RBC)	4.34 x 10^9^/L	4.7-6.1 x 10^9^/L
Hemoglobin	12.8 g/dL	12-15 g/dL (women)
Hematocrit	38%	36%-47% (women)
Mean corpuscular volume (MCV)	89.5 fL	80-100 fL
Mean corpuscular hemoglobin (MCH)	29.5 pg/cell	27 to 31 pg/cell
Platelets	337 x 10^9^/L	150-400 x 10^9^/L
Sodium	139 mmol/L	135-147 mmol/L
Potassium	2.8 mmol/L	3.5-5.0 mmol/L
Chloride	101 mmol/L	98-106 mmol/L
Random glucose	5.36 mmol/L	5.5–11 mmol/L
Urea level	2.8 mmol/L	3.6-7.1 mmol/L
Amylase	53 units/L	30-220 units/L
Calcium	2.2 mmol/L	2.0-2.5 mmol/L
Phosphorus	1.2 mmol/L	0.97-1.45 mmol/L
Creatinine	55.8 μmol/L	44-97 μmol/L
Magnesium	0.47 mmol/L	0.65-1.05 mmol/L
CK-MB	17 units/L	30-135 units/L
Troponin-I	0.00 ng/mL	< 0.03 ng/mL
Spot urine sodium	26 mEq/L	>20 mEq/L
Spot urine chloride	139 mEq/L	<20 mEq/L
Spot urine potassium	29 mEq/L	<20 mEq/L
24-hour urine potassium	68.2 mEq/L	< 30 mEq/L
Repeated 24-hour urine potassium	110.2 mEq/L	< 30 mEq/L
24-hour urine calcium	0.0	Females: 20-275 mg/24-hour specimen
Urine pH	8	4.5-8
Serum pH	7.49	7.35-7.45
Serum bicarbonate	29.2 mEq/L	21-28 mEq/L
Arterial blood partial pressure of carbon dioxide	33.5 mmHg	35-45 mmHg
Post-treatment potassium	3.6mmol/L	3.5-5.0 mmol/L
Post-treatment magnesium	0.7mmol/L	0.65-1.05 mmol/L

## Discussion

The kidney's processing of ions, such as sodium, chloride, magnesium, calcium, and potassium, is a complex process that is dependent on the molecular activity of numerous renal tubular channels. GS is a genetic tubulopathy characterized by hypokalemic metabolic alkalosis, hypocalciuria, hypomagnesemia, activated Renin-Angiotensin System (RAS), and high Angiotensin II levels caused by loss-of-function mutations in the SLC12A3 gene, which encodes the Na+-Cl cotransporter [3]. However, the cardiovascular effects of angiotensin II are muted because GS patients are either normotensive or hypotensive, representing a type of endogenous angiotensin II antagonism [4,5]. GS is similar to the disruption created by thiazide diuretics (inhibition of the NaCl cotransporter in the distal convoluted tubule of the nephron), suggesting that the NCC/SLC12A3 gene could be a candidate [3]. Despite the increasing number of confirmed causal mutations, up to 40% of patients are discovered to possess only one SLC12A3 mutant allele; thus, extensive genomic rearrangement could account for undiscovered mutations [6]. GS patients can present with diverse complaints and manifestations including salt craving, episodes of fainting, muscle weakness, dizziness fatigue, tetany, cramps, carpopedal spasms, paraesthesia, growth retardation, thirst, polyuria, nocturia, and palpitations [5]. However, most of the patients have subtle clinical findings or unremarkable physical examinations. The following are the suggested biochemical criteria for identification of GS: Low serum potassium level (hypokalemia), low serum magnesium level (hypomagnesemia), high serum bicarbonate level (metabolic alkalosis), high plasma renin and aldosterone levels, with low urinary calcium excretion (hypocalciuria) (Table 2) [5,7]. GS is typically treated with a high salt intake, as well as magnesium and potassium supplements that are taken orally. It is critical to use potassium chloride rather than alternative salts containing poorly absorbable anions such as aspartate or gluconate as these may aggravate the underlying metabolic alkalosis and do not correct hypokalemia [5]. Magnesium replacement using magnesium chloride is the treatment of choice for hypomagnesemia given at a daily dose of 4-5 mg/kg per day divided into four to six doses to avoid diarrhea [8]. Potassium-sparing diuretics and aldosterone antagonists are considered in the treatment of normotensive GS patients. However, these medications should be used with caution in patients with low blood pressure as they might worsen the condition [9]. In general, GS has a great long-term prognosis. The severity of fatigue, on the other hand, may substantially impair some patients' everyday activities. Renal insufficiency is exceptionally rare in GS [2]. In this paper, we discussed a challenging case of persisting hypokalemia in a 30-year-old woman who was complaining of generalized fatigue, palpitation, and limb numbness for the last eight years without being diagnosed or managed appropriately. What made her symptoms more apparent was the episode of enteritis she had, which increased her extra-renal electrolytes loss and made her seek medical care for her diarrheal illness and other symptoms. As a result, when a patient presents with persistent, unspecified, and inadequately treated hypokalemia, tests for GS should indeed be considered.

**Table 2:** The proposed biochemical criteria for suspecting a diagnosis of GS The table is adapted from [5]. GS - Gitelman syndrome

Criteria for supporting the diagnosis of GS	Criteria against the diagnosis of GS
Chronic hypokalemia (<3.5 mmol/L) with inappropriate renal potassium wasting (spot potassium-creatinine ratio >2.0 mmol/ mmol [>18 mmol/g])	Absence of hypokalemia (except in the case of renal failure)
Hypomagnesemia (<0.7 mmol/l) with inappropriate renal magnesium wasting	Low urinary potassium excretion (spot potassium-creatinine ratio <2.0 mmol/mmol; hypercalciuria
Hypocalciuria (spot urine, calcium-creatinine ratio <0.2 mmol/mmol)	Use of thiazide diuretics or laxatives
Normal or low blood pressure	Hypertension
High renin activity	Low renin activity
Fractional excretion of chloride >0.5%	Family history of kidney disease that is inherited in an autosomal dominant manner.
Normal renal ultrasound with the absence of renal abnormalities or nephrocalcinosis	Nephrocalcinosis, unilateral kidney, nephrolithiasis, or cystic kidneys
Metabolic alkalosis	Absence of metabolic alkalosis

## Conclusions

Renal potassium wasting, hypokalemia, metabolic alkalosis, hypocalciuria, hypomagnesemia, and hyperreninemic hyperaldosteronism are all biochemical features of GS, an autosomal recessive salt-losing tubulopathy. For clinicians who are not experts in this field, diagnosing GS is extremely difficult and challenging. In addition, clinicians face a challenge in maintaining electrolyte balance, preventing complications, and improving prognosis with long-term diseases such as GS. We have made a clinical diagnosis of GS in a 30-year-old woman with hypokalaemia, hypomagnesemia, and other typical symptoms of GS. Despite a solid understanding of the disease, the wide spectrum of clinical manifestations and overlapping features with other causes of electrolyte abnormality makes the diagnosis of GS a challenging one.
